# Antiaging Effect of 4-*N*-Furfurylcytosine in Yeast Model Manifests through Enhancement of Mitochondrial Activity and ROS Reduction

**DOI:** 10.3390/antiox11050850

**Published:** 2022-04-26

**Authors:** Paweł Pawelczak, Agnieszka Fedoruk-Wyszomirska, Eliza Wyszko

**Affiliations:** Institute of Bioorganic Chemistry, Polish Academy of Sciences, Noskowskiego 12/14, 61-704 Poznań, Poland; ppawelczak@ibch.poznan.pl

**Keywords:** cytosine derivative, pyrimidine derivative, antiaging, budding yeast, chronological lifespan, mitochondria, antioxidant

## Abstract

Small compounds are a large group of chemicals characterized by various biological properties. Some of them also have antiaging potential, which is mainly attributed to their antioxidant activity. In this study, we examined the antiaging effect of 4-*N*-Furfurylcytosine (FC), a cytosine derivative belonging to a group of small compounds, on budding yeast *Saccharomyces cerevisiae.* We chose this yeast model as it is known to contain multiple conserved genes and mechanisms identical to that of humans and has been proven to be successful in aging research. The chronological lifespan assay performed in the study revealed that FC improved the viability of yeast cells in a concentration-dependent manner. Furthermore, enhanced mitochondrial activity, together with reduced intracellular ROS level, was observed in FC-treated yeast cells. The gene expression analysis confirmed that FC treatment resulted in the restriction of the TORC1 signaling pathway. These results indicate that FC has antiaging properties.

## 1. Introduction

Low-molecular-weight compounds represent a substantial group of bioactive chemicals, which are widely used, for example, in anticancer therapies [1], the treatment of autoimmune, inflammatory, and related diseases [2,3,4], and the management of neurodegenerative disorders, such as Parkinson’s [5,6,7] and Alzheimer’s [8] disease. Their biological effect is mainly attributed to their antioxidant activity [3]. According to the free radical theory [9] and the mitochondrial theory of aging [10], reactive oxygen species (ROS) play a crucial role in the aging process. Both theories state that the extensive generation of ROS leads to oxidative stress, contributing to unfavorable cellular events, including protein and DNA oxidation and mitochondrial dysfunction [11]. These in turn lead to the loss of cellular function and ultimately cell death [12]. 

Compounds that can reduce ROS and the associated effects of oxidative stress may act as antiaging agents by preventing or mitigating the age-related malfunction of senescent cells [13]. Since aging is a growing concern in modern society, researchers have been focusing on finding molecules capable of promoting healthy aging and extending healthspan [12]. Several research models have been established for aging, which is a well-conserved process among eukaryotes. One of the most important models is the well-studied budding yeast *Saccharomyces cerevisiae*. Yeast is a unicellular, rapid-growing organism which shares conserved signaling pathways and genes with humans [14,15]. Thus, it is considered as an appropriate model to study not only aging but also accompanying processes such as oxidative stress resistance, mitochondrial activity, and cellular damage [12,15]. The limitations of this model include the fact that it is different from mammals’ metabolic and reproductive strategies, namely fermentative metabolism in aerobic conditions and asymmetric divisions, generating mother and daughter cells.

Over the years, two yeast aging models have been developed. One is the replicative lifespan (RLS) model, which measures the number of divisions that a cell can complete before death, and the other is the chronological lifespan (CLS) model, which assesses the viability of cell culture in time. RLS is thought to represent an aging model for dividing cells in higher eukaryotes, and CLS as a model corresponds to postmitotic cells [16]. Several antiaging compounds, including resveratrol, rapamycin, and metformin, have been characterized using the yeast model. These compounds affect different molecular pathways, and their effectiveness has been confirmed in higher eukaryotes [17]. The extension of lifespan is usually associated with the management of stress or metabolic shift [13]. The processes that affect lifespan in yeast and higher eukaryotes are the generation of ROS, the induction of autophagy, calorie restriction (CR) through the TORC1 or AMPK signaling pathway, and the activation of sirtuins [17]. 

4-*N*-Furfurylcytosine (FC) is a cytosine derivative modified with furfural at the N4 position. The biological activity and clinical significance of purine and pyrimidine derivatives are well documented. Among these compounds, cytosine arabinoside (cytarabine) is the most widely used in the treatment of leukemias (WHO). Another compound, 6-Furfuryladenine (kinetin), has been shown to promote healthy aging [18], while *N*-6-Furfuryladenosine (kinetin riboside) and its derivatives were proven to be selective anticancer agents [19]. Preliminary results obtained from our experiments with human cell lines (unpublished data) indicated that FC exhibits promising biological activities, such as the stimulation of proliferation, ROS scavenging, or the enhancement of mitochondrial function, which together could have a potential antiaging effect. 

In this study, we investigated the effect of FC on the viability and growth of *S. cerevisiae* yeast, as well as the bioenergetic status of cells manifested by respiration rates and mitochondrial membrane potential (MMP) under both normal and oxidative stress conditions. To understand the mechanism of action of FC in detail, we estimated the expression levels of several genes associated with cell growth, respiration, and oxidative stress resistance. The results obtained from flow cytometry, confocal microscopy, respirometry, and real-time polymerase chain reaction (real-time PCR or qPCR) analyses substantially corroborated the potential antiaging properties of FC.

## 2. Materials and Methods

### 2.1. 4-N-Furfurylcytosine

4-*N*-Furfurylcytosine used in the study was synthesized in the Institute of Bioorganic Chemistry of the Polish Academy of Sciences (Poznań, Poland). 

### 2.2. Yeast Strain and Media

Yeast *Saccharomyces cerevisiae* strain BY4741 (MATa, his3Δ1, leu2Δ0, met15Δ0, and ura3Δ0) used in all experiments was kindly provided by dr Kamilla Grzywacz (Institute of Bioorganic Chemistry, Polish Academy of Sciences, Poznań, Poland). The cultures were initiated by inoculating frozen stock cultures onto solid YPD plates (1% yeast extract, 2% peptone, 2% glucose, and 2% agar). Then, single colonies from YPD plates were inoculated onto YPD or YPG (1% yeast extract, 2% bacto-peptone, and 2% glycerol) liquid medium.

### 2.3. CLS Analysis

Yeast cells were grown in YPD medium supplemented with 0.25, 0.5, or 1.0 mM FC at 30 °C with constant shaking (180 rpm). Starting on day 3, 100 μL of cell suspension was harvested every 2–3 days via centrifugation (14,000 rpm, 5 min). Cells were washed twice with DPBS (Thermo Fisher Scientific, Waltham, MA, USA), resuspended at a final concentration of 7.5 μg/μL in 500 μL of DPBS containing propidium iodide (PI; Sigma-Aldrich, St. Louis, MO, USA; ex/em: 535/617 nm), and incubated for 30 min at 30 °C in the dark. Immediately after staining, cells were analyzed using a FACSCalibur flow cytometer (Becton Dickinson, Franklin Lakes, NJ, USA) at an excitation wavelength of 488 nm. The obtained data were analyzed using FlowJo software (Becton Dickinson, Franklin Lakes, NJ, USA).

### 2.4. Growth Kinetics

Exponentially growing yeast cells were inoculated onto YPD medium supplemented with 0.25, 0.5, or 1.0 mM FC. The cultures were grown for 10 h at 30 °C with shaking (180 rpm). The optical density (OD) of the growing cultures was measured at different time-points at a wavelength of 600 nm using an Eppendorf Biophotometer 6131.

### 2.5. Flow Cytometry Analyses

#### 2.5.1. Effect of FC on MMP

Yeast cells were cultured in YPG medium supplemented with 0.1, 0.25, 0.5, 0.75, or 1.0 mM FC overnight at 30 °C with shaking (180 rpm). Then, cells were harvested via centrifugation (14,000 rpm, 5 min), washed twice with DPBS, resuspended in DPBS containing 180 nM tetramethylrhodamine and ethyl ester (TMRE; Invitrogen, Waltham, MA, USA; ex/em: 549/574 nm), and incubated for 30 min at 30 °C in the dark. Immediately after staining, cells were analyzed using a FACSCalibur flow cytometer (Becton Dickinson, Franklin Lakes, NJ, USA) at an excitation wavelength of 488 nm. The obtained data were analyzed using FlowJo software (Becton Dickinson, Franklin Lakes, NJ, USA).

#### 2.5.2. Protective Effect against H_2_O_2_

Yeast cells were grown in YPG medium supplemented with 0.5 mM FC overnight at 30 °C with shaking (180 rpm). Then, 1 mL of cell suspension was transferred onto 12-well plates and incubated for 2 h with 1, 2, 3, 5, or 10 mM hydrogen peroxide (H_2_O_2_). After incubation, cells were harvested, stained with TMRE, and analyzed as described before.

#### 2.5.3. Regenerative Effect after H_2_O_2_ Treatment

Yeast cells were cultured in YPG medium overnight at 30 °C with shaking (180 rpm). Then, 1 mL of culture was transferred onto 12-well plates and incubated with 1, 2, 3, 4, or 5 mM H_2_O_2_ for 1 h. After incubation, cells were harvested via centrifugation (5000 rpm, 5 min), resuspended in fresh YPG medium supplemented with 0.5 mM FC, and incubated for 2 h at 30 °C with shaking. Then, cells were harvested, stained with TMRE, and analyzed as described before.

#### 2.5.4. ROS Measurement

Yeast cells were grown in YPD or YPG medium supplemented with 0.25, 0.5, or 1.0 mM FC overnight at 30 °C with shaking (180 rpm). Next, cells were harvested via centrifugation (14,000 rpm, 5 min), rinsed twice with DPBS, resuspended in DPBS containing 10 μM of 2′,7′-dichlorodihydrofluorescein diacetate (H2DCFDA; Thermo Fisher Scientific, Waltham, MA, USA; ex/em: ~492–495/517–527 nm), and incubated for 30 min in the dark. Immediately after staining, cells were analyzed using a FACSCalibur flow cytometer (Becton Dickinson, Franklin Lakes, NJ, USA) at an excitation wavelength of 488 nm. The obtained data were analyzed using FlowJo software (Becton Dickinson, Franklin Lakes, NJ, USA).

### 2.6. Confocal Microscopy Analyses

Yeast cells were cultured in YPG medium supplemented with 0.25, 0.5, or 1.0 mM FC at 30 °C with shaking (180 rpm) and harvested at an appropriate time-point (detailed below). Next, cells (100 µL) were centrifuged (14,000 rpm, 5 min), washed twice in DPBS, and resuspended in 500 µL of fresh DPBS. Finally, cells were stained and analyzed using a Leica TCS SP5 confocal laser scanning microscope. Image processing was controlled using Leica LAS AF 2.7.3 software. Fluorescence intensity and size were analyzed using Leica LAS X 3.3.3 software with 2D or 3D deconvolution modules.

#### 2.6.1. FC Effect on the Yeast Cell Viability

To analyze the viability, yeast cells were harvested on day 14 of the CLS analysis and stained in 500 µL of DPBS containing 7.5 µg/µL PI for 30 min and 10 µg/mL Hoechst 33342 for 15 min at 30 °C with shaking (350 rpm). Next, cells were washed with DPBS to remove excess dyes and resuspended in 50 µL of DPBS, and then, 2–3 µL of cell suspension was placed on microscope slides coated with concanavalin A (2 mg/mL). Live-cell imaging was performed using HC PL APO 63×/1.4 oil-immersion objective with 2x digital zoom. Sequentially scanned images were collected at an excitation/emission wavelength of 405/435–480 nm for living cells (blue, Hoechst 33342) and 543/585–640 nm for dead cells (red, PI).

#### 2.6.2. Mitochondrial Membrane Potential

For the confocal microscopy analysis of the changes in MMP, yeast cells were harvested after 24 h of FC treatment and stained in 50 µL of DPBS containing 180 nM TMRE for 30 min at 30 °C with shaking (350 rpm). Next, cells were washed, and 2–3 µL of cell suspension was placed on microscope slides coated with concanavalin A (2 mg/mL). Images in Z-stack were collected using HC PL APO 63×/1.4 oil-immersion objective with 2.7x digital zoom at an excitation/emission wavelength of 543/570–610 nm. A *Z*-projection of the images was created from Z-stacks using the “max” intensity option of LAS X 3.3.3 software, and then, the regions of interest (ROIs) were selected to measure the fluorescence intensity of each yeast cell. The results are presented as the mean of fluorescence intensity.

#### 2.6.3. Comparative Analysis of Maturity and Cell Size after FC Treatment

The maturity and size of cells were determined based on bud scars and MMP. Briefly, yeast cells were harvested on day 14 of the CLS analysis and then simultaneously stained in 500 µL of DPBS containing 50 μg/mL wheat germ agglutinin (WGA) Alexa Fluor 488 conjugate (Invitrogen, Waltham, MA, USA) for 45 min and 180 nM TMRE for 30 min at 30 °C with shaking (350 rpm). Z-stack live-cell imaging was performed using HC PL APO 63×/1.4 oil-immersion objective with 2 × digital zoom. Images were sequentially scanned at an excitation/emission wavelength of 488/500–560 nm for green fluorescence (bud scars) and 543/570–610 nm for red fluorescence (MMP). The analysis was performed based on the ROI area. *Z*-projection was created using the “max” intensity option of LAS X 3.3.3 software, and then, the ROIs were selected to measure the red and green fluorescence intensity of each yeast cell.

### 2.7. Respiration Rate Analysis

Yeast cells were grown in YPG or YPD medium overnight. Then, 1 mL of cell suspension was inoculated onto a fresh medium supplemented with 1.0 mM FC and grown overnight. The respiration analysis was performed as described by Karachitos et al. [20] with slight modifications as follows: Cells were harvested via centrifugation (5000 rpm, 5 min), washed once, and resuspended in PBS. The oxygen uptake of 1 or 4 OD units of cells was measured using a Clark-type oxygen electrode (Oxygraph+ system, Hansatech Instruments Ltd., Pentney, UK) in 1 mL of YPG or YPD medium at 30 °C, respectively. To induce state 4 and state U, 10 μM tributylin (TBT; Sigma-Aldrich, St. Louis, MO, USA) and 3 μM carbonyl cyanide p-trifluoromethoxy phenylhydrazone (FCCP; Sigma-Aldrich, St. Louis, MO, USA) were added, respectively. The working concentrations of TBT and FCCP were verified experimentally. After recording, the protein concentration of cells was estimated. Briefly, cells were centrifuged, incubated with 100 U of lyticase and 10 mM DTT (A&A Biotechnology, Gdynia, Poland) at 30 °C for 30 min, and then lysed via three freeze–thaw cycles. The obtained lysates were centrifuged, cellar debris was discarded, and the protein concentration was measured spectrophotometrically at 280 nm. The following respiration parameters were determined: Basal respiration indicated the actual oxygen consumption by cells and fluctuated between a TBT-sensitive resting state (state 4) and phosphorylating state (state 3), which is the difference between basal respiration and state 4. The uncoupling state (state U) corresponded to maximal respiration and was evaluated using FCCP titration. The mitochondrial coupling efficiency and mitochondrial coupling capacity were calculated as a ratio of state 3 to basal respiration and state U to state 4, respectively. The spare respiratory capacity (SRC) was defined as the difference between maximal and basal respiration. The obtained results were normalized to 1 mg of protein.

### 2.8. Real-Time PCR

#### 2.8.1. Total RNA Isolation

Yeast cells were grown overnight in the presence of 0.5 or 1.0 mM FC. Then, cells were collected via centrifugation and immediately frozen in liquid nitrogen. Before RNA isolation, cells were incubated with 100 U of lyticase and 10 mM DTT (A&A Biotechnology, Gdynia, Poland) at 30 °C for 30 min. The resulting spheroplasts were centrifuged (3000 rpm, 5 min), and total RNA was isolated using EXTRAzol (BLIRT S.A., Gdańsk, Poland) according to the manufacturer’s protocol. DNA residues were removed by adding DNase I (DNA-free DNA Removal Kit; Thermo Fisher Scientific, Waltham, MA, USA). The total RNA concentration was measured using a NanoDrop 2000 UV/Vis spectrophotometer at 260 nm.

#### 2.8.2. cDNA Synthesis and qPCR

Reverse transcription was performed using 500 ng of isolated RNA and a Transcriptor First-Strand cDNA Synthesis Kit (Roche, Basel, Switzerland) according to the manufacturer’s protocol. Random hexamer primers were used for cDNA synthesis. Real-time PCR analysis was performed as described [5]. The expression levels of CCS1 (Saccharomyces Genome Database, SGD ID: S000004641), ECL1 (SGD ID: S000003378), FOB1 (SGD ID: S000002517), GTR1 (SGD ID: S000004590), HAP4 (SGD ID: S000001592), RIM15 (SGD ID: S000001861), SCH9 (SGD ID: S000001248), and SOD2 (SGD ID: S000001050) genes were determined. The primers used in the analysis were designed using ProbeFinder Software (Roche, Basel, Switzerland) and are listed in Table 1. Each cDNA sample was analyzed using Mono Color Hydrolysis UPL Probes (Roche, Basel, Switzerland). The PCR mixtures were prepared as per the manufacturer’s instructions. The PCR conditions applied for the amplification of all studied genes were as follows: initial incubation at 94 °C for 10 min, followed by 45 cycles of amplification (15 s at 94 °C, 30 s at 60 °C, and 15 s at 72 °C) (single acquisition), and a final cooling step at 40 °C for 2 min. Analysis was performed using LightCycler 480 II (Roche, Basel, Switzerland). Relative gene expression was calculated using the Roche Applied Science E-Method and normalized to that of the reference genes ACT1 (SGD ID: S000001855), ALG9 (SGD ID: S000005163), TAF10 (SGD ID: S000002574), TFC1 (SGD ID: S000000327), and UBC6 (SGD ID: S000000902). All standard curves were generated by amplifying a series of twofold dilutions of cDNA.

### 2.9. Statistical Analysis

Each experiment consisted of at least two biological and three technical replicates. Statistical analyses were performed using GraphPad Prism 8.0.1 for Windows (GraphPad Software, San Diego, CA, USA). CLS was analyzed using two-way analysis of variance (ANOVA) followed by Tukey’s multiple comparison test. ROS level and MMP were analyzed using one-way ANOVA followed by Dunnett’s multiple comparison test. The regenerative and protective potential of FC against H_2_O_2_ was investigated using two-way ANOVA followed by Sidak’s multiple comparison test. Cell size was analyzed using the Kruskal–Wallis test. The significance of respiration rates was evaluated using Student’s *t*-test. The level of gene expression in three independent biological replicates (each with three experimental repeats) was analyzed using one-way ANOVA followed by Dunnett’s multiple comparison test. A *p*-value of <0.05 was considered statistically significant.

## 3. Results

### 3.1. FC Supplementation Extends Yeast Survival

**Figure 1:**
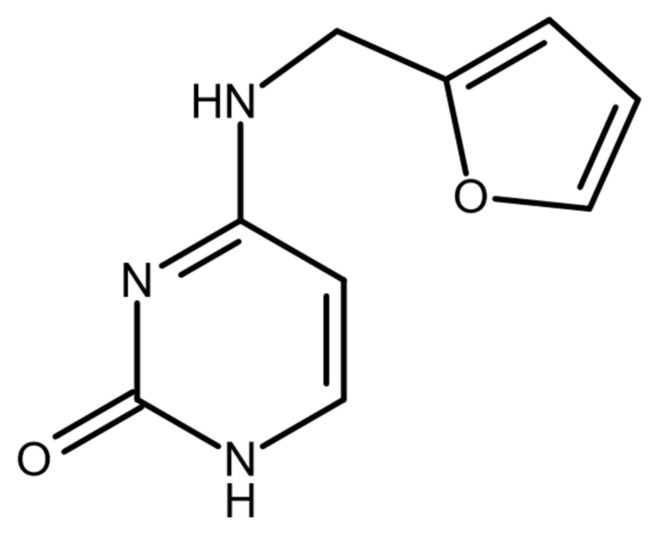
Chemical structure of FC.

The aim of this study was to investigate the antiaging and proproliferative properties of FC (Figure 1). For this purpose, we analyzed the effect of FC on yeast growth and physiology, including mitochondrial performance and ROS generation as well as sensitivity. 

**Figure 2:**
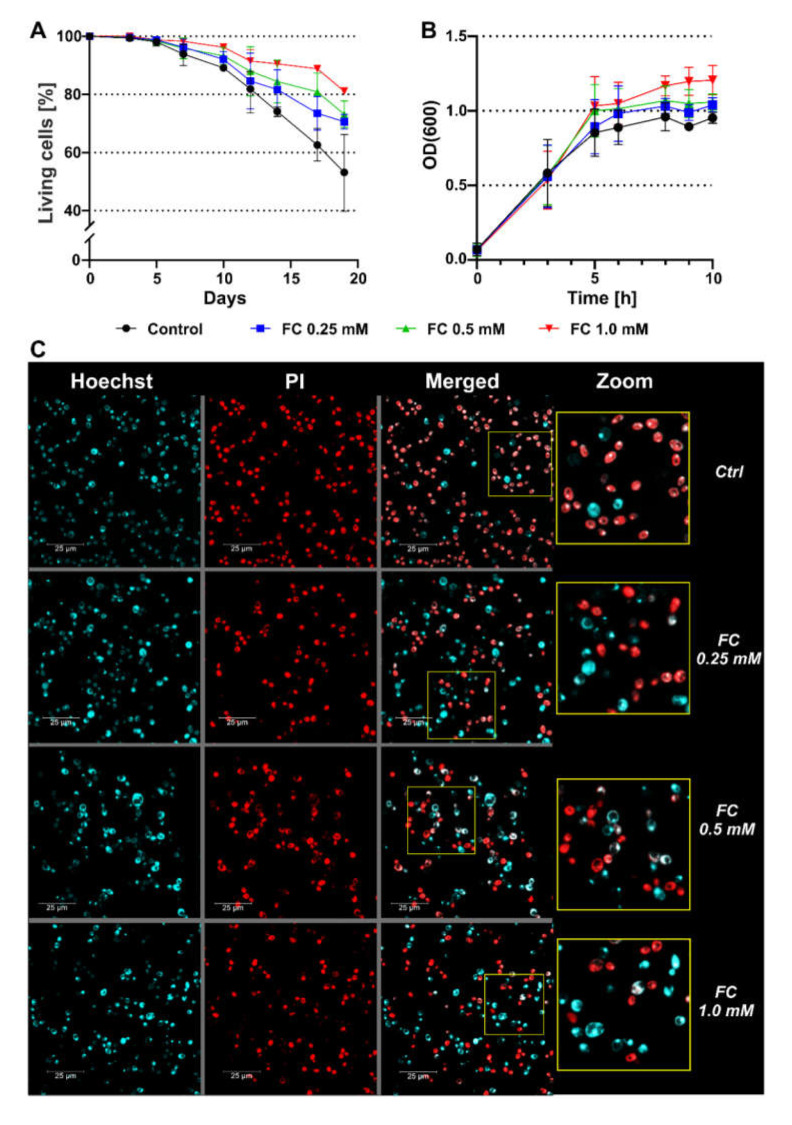
Effect of FC supplementation on the viability of BY474 yeast cells. (**A**) Yeast CLS in the presence of 0.25 (blue ■), 0.5 (green ▲), or 1.0 (red ▼) mM FC, and in its absence (control, black ●), evaluated using flow cytometry using PI staining. Since PI enters dead cells, unstained cells were considered alive. Data are presented as the mean percentage of live cells within the total analyzed population (10,000 cells) ± SD of three independent experiments. (**B**) Growth kinetics of yeast in the exponential phase determined based on OD_600_ measurements. Data are presented as mean ± SD of three independent experiments. (**C**) Confocal microscopy imaging of cells on day 14 of the CLS assay using Hoechst 33342 and PI staining. Blue (ex/em: 405/435–480 nm) and red (ex/em: 543/585–640 nm) fluorescence correspond to live and dead cells, respectively. Merged images are shown on the right panels.

In the first step, the influence of FC on yeast viability was investigated by performing a CLS assay of *S. cerevisiae* wild-type strain BY4741. The analysis was carried out in optimal growth conditions in glucose-rich complete medium (YPD) at 30 °C. CLS is an established model of yeast aging, in which cell survival is tracked in the stationary phase [21]. Based on flow cytometry and staining with PI, a dye that only binds to the DNA of permeable cells, due to which stained cells are considered dead, we found that FC supplementation prolonged the lifespan of yeast. As shown in Figure 2A, FC dose-dependently enhanced the viability of yeast throughout the experiment. Our results showed that 1.0 mM was the most effective concentration of FC at which statistically significant differences (versus control) were observed even on day 12 of the CLS assay (over 10% more viable cells), whereas groups treated with 0.5 and 0.25 mM FC only showed statistically significant differences on days 14 and 17, respectively (Table 2). However, within the experimental groups, significant differences were only noted between groups treated with 0.25 and 1.0 mM FC on days 17 and 19. Day 19 was the last day of the CLS assay, and at this time-point, we observed over 50% higher numbers of viable yeast cells which were grown in medium supplemented with 1.0 mM FC in comparison with the control.

The extension of CLS may be caused by a reduction in growth rate, which results in the delayed initiation of the stationary phase and nutrient depletion. To verify this hypothesis, we assessed the growth kinetics of yeast in the exponential phase using the OD measurements recorded at a wavelength of 600 nm (OD_600_), under the same conditions as those applied in the CLS assay. However, the obtained results contradicted this hypothesis, even suggesting the enhanced growth of treated groups, but the differences were not statistically significant (Figure 2B). Nevertheless, we did not observe the inhibition of yeast growth with any of the tested concentrations of FC. 

Day 14 of the CLS assay was the first time-point at which significant differences were observed for the two highest concentrations of FC (1.0 and 0.5 mM) and a positive trend for the lowest (0.25 mM) applied concentration, as compared with the control. Therefore, on this day, we analyzed yeast viability using confocal microscopy by double-staining the cells with Hoechst and PI (Figure 2C). While PI can only penetrate permeable cells (red fluorescence), Hoechst binds to DNA (blue fluorescence) regardless of the cell membrane status. Thus, red cells are considered dead and blue cells as viable. In line with the results obtained from flow cytometry analysis, we observed more blue-stained and less red-stained cells in the samples treated with FC, which indicated that the viability of FC-treated cells was higher on day 14 of the CLS assay, and the effect of FC on viability was dose-dependent. 

**Figure 3:**
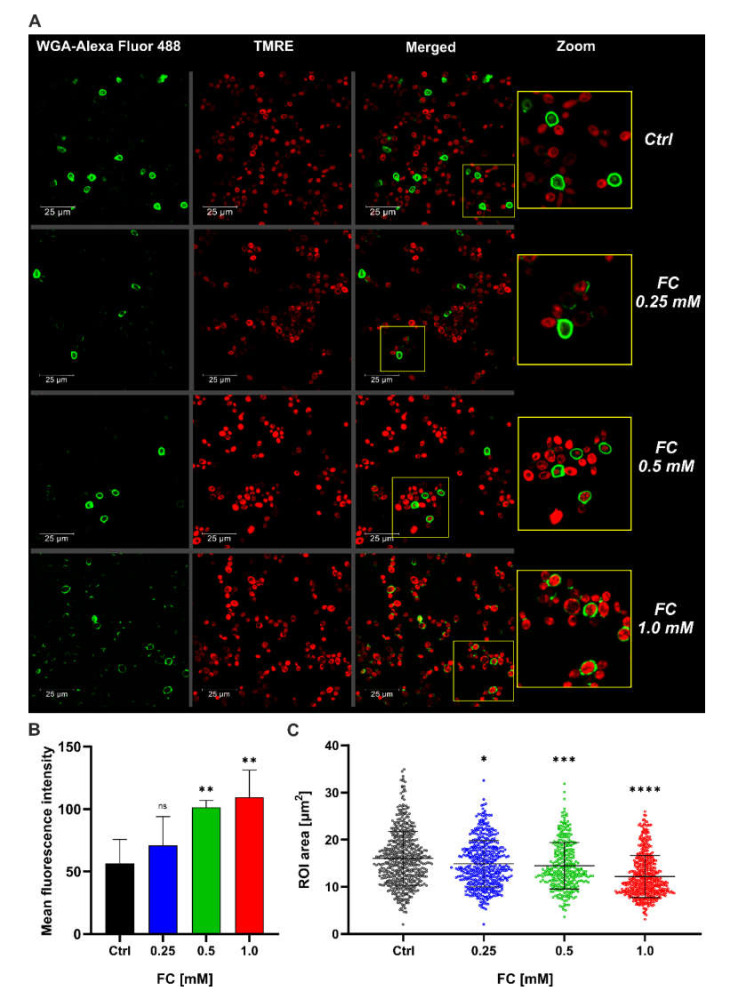
Analysis of FC influence on the mitochondrial membrane potential and cell size of aged yeast (day 14 of CLS assay). (**A**) Confocal microscopy analysis of yeast maturity and cell size. Intensity of TMRE red fluorescence corresponds to the energetic state of mitochondria (ex/em: 543/570–610 nm). Green fluorescence of WGA-Alexa Fluor 488 indicates bud scars (ex/em: 488/500–560 nm). (**B**) Bar graph presents changes in mean fluorescence intensity of TMRE. Data are presented as mean ± SD of three independent experiments. (**C**) Size of yeast cells evaluated using the ROI area. Number of samples: Ctrl = 544; 0.25 mM FC = 485; 0.5 mM FC = 342; 1.0 mM FC = 541. Statistical significance versus control (ANOVA): (ns) non−significant, (*) *p* < 0.05, (**) *p* < 0.001, (***) *p* < 0.001, (****) *p* < 0.0001.

We further examined the cells on that CLS time-point using confocal microscopy after staining with TMRE and WGA conjugated with Alexa Fluor 488. TMRE is a cationic dye that targets active mitochondria and accumulates in proportion to ΔΨm emitting red fluorescence and thus allows changes in MMP to be monitored. WGA is a lectin that binds to N-acetyl-glucosamine residues, which are the major component of *S. cerevisiae* bud scars and occur with the division of every yeast cell, thus acting as an indicator of yeast replicative age [22]. WGA-Alexa Fluor 488 stains the cell wall structures green. As shown in Figure 3A, in groups treated with 1.0 and 0.5 mM FC, we observed higher TMRE fluorescence intensity, indicating higher MMP as compared to the control. Moreover, in the presence of FC, we noted not only fewer “old cells” (cells with cell walls completely stained by WGA) but also fewer cells exhibiting intense TMRE signals, which suggests high mitochondrial membrane potential. This effect was more pronounced with increasing concentrations of FC. On the other hand, in the control, we did not observe TMRE fluorescence in “old cells,” and these cells were higher in number. We also estimated cell size by calculating the ROI area of each yeast cell in confocal microscopy images (Figure 3B). The cell size decreased with increasing concentrations of FC. The average cell area in the control was approximately 16 μm^2^, while in the groups supplemented with 1.0 mM FC, it was 12.18 μm^2^, indicating a reduction of about 25%. In yeast supplemented with 0.25 and 0.5 mM FC, the difference in comparison with the control was about 10%. Taken together, on day 14 of the CLS assay, FC-treated yeast cells exhibited higher MMP than the control, and the effect of FC was dose-dependent. In FC-treated cultures, the replicatively oldest cells preserved high MMP, while in the control, the cells displayed low MMP. These results suggest that the enhancement of yeast viability via FC is related to its positive effect on the mitochondrial energetic state. 

### 3.2. FC Strongly Enhances Mitochondrial Performance in Glucose Medium and Also Reduces ROS Level

**Figure 4:**
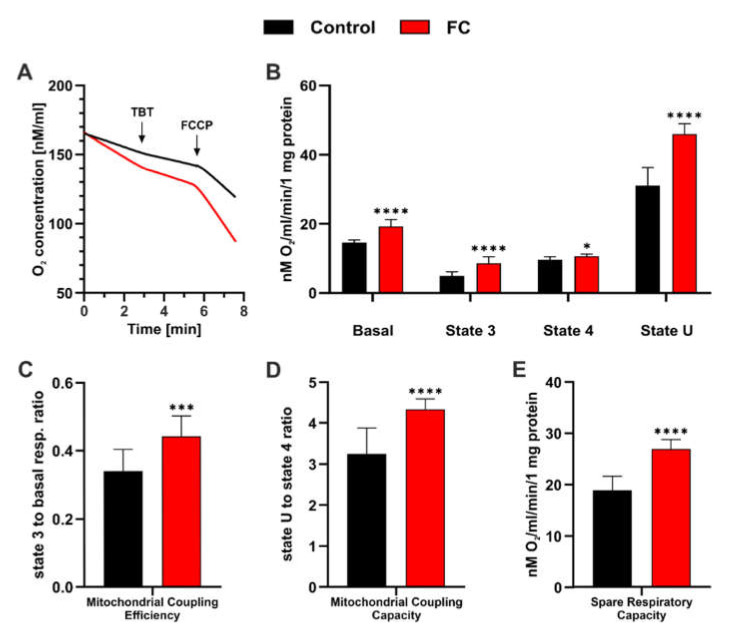
Effect of FC supplementation on the energy status of yeast cells growing in YPD medium. Recording was performed in YPD medium using the Oxygraph+ system. (**A**) Representative recordings of O_2_ consumption by yeast cells. (**B**) Respiratory states calculated for control (black bars) and FC-treated (red bars) yeast cells. Basal respiration indicates actual oxygen consumption by cells, which fluctuates between phosphorylating state (state 3) and resting state (state 4). State 4 was determined using TBT titration, while state 3 was determined as the difference between basal respiration and state 4. Uncoupling state (state U), which corresponds to maximal respiration, was evaluated using FCCP titration. (**C**) Contribution of state 3 to basal respiration, which corresponds to mitochondrial coupling efficiency. (**D**) Mitochondrial coupling capacity calculated as a ratio of state U and state 4. (**E**) SRC defined as the reserve between basal and maximal respiration. Data are presented as mean ± SD of three independent experiments. Statistical significance versus control (*t*-test): (*) *p* < 0.05, (***) *p* < 0.001, (****) *p* < 0.0001.

In the next step, we examined the bioenergetic status of intact yeast cells using the Oxygraph+ system. For this analysis, we chose the highest FC concentration (1.0 mM), as it was identified as the most effective in the previous experiment, and applied the same environmental conditions. Figure 4A shows the representative traces of respiration measurements used for further calculations, as described in Section 2.7. The rate of oxygen consumption by yeast cells cultivated in the presence of FC was clearly higher than that by control cells, as indicated by the basic respiratory states derived from the recordings and presented in Figure 4B. The marked increase in basal respiration (~30%) was mainly due to an over 70% increase in the level of phosphorylating state (state 3), whereas resting state (state 4) only increased by 10%. However, significant enhancement, of up to 50%, was also observed in maximal respiration (state U). This implies that in FC-treated cells, basal respiration accounted for about 41% of maximal respiration, while in the control, it was about 48%. Since state 3 indicates the level of mitochondrial ATP synthesis and state 4 corresponds to the rate of proton leak across the mitochondrial membrane, the results suggest that FC-treated cells are characterized by increased mitochondrial ATP demand and high respiratory efficiency. Indeed, as shown in Figure 4C, mitochondrial coupling efficiency was significantly higher (~30%) in FC-treated cells, and interestingly, it was almost similar to the level of control cells grown in glycerol medium (Figure S1). The calculated mitochondrial coupling capacity (Figure 4D) and SRC (Figure 4E) were higher by over 30% and 40%, respectively, which confirm high mitochondrial quality and potential. These findings suggest that FC treatment forced a metabolic switch toward oxidative phosphorylation, together with a definite enhancement of mitochondrial capability, in yeast cells.

**Figure 5:**
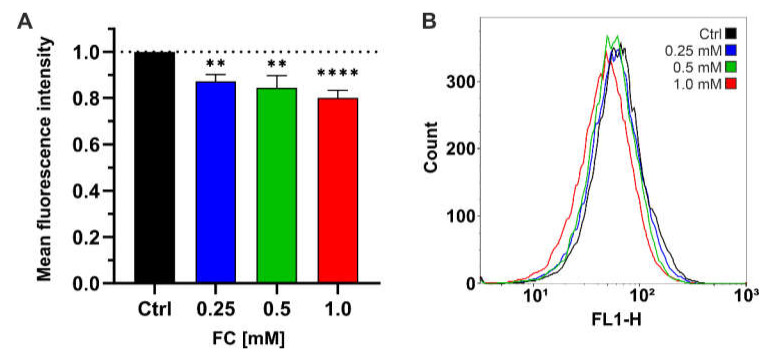
Flow cytometry analysis using H_2_DCFDA staining for the determination of ROS content in control cells and cells treated with 0.25, 0.5 mM, or 1.0 mM FC. Bar graph (**A**) presents relative mean fluorescence intensity ± SD, whereas histogram (**B**) presents the representative data from three independent experiments. Data are presented as mean ± SD of three independent experiments. Statistical significance versus control (ANOVA): (**) *p* < 0.001, (****) *p* < 0.0001.

Oxidative metabolism results in the generation of superoxide anions (O_2_^•−^) as a by-product of respiration, which makes mitochondria one of the primary sources of ROS. The accumulation of ROS contributes to mitochondrial and cellular damage, promoting both replicative and chronological aging in yeast [23,24]. However, the respiratory chain prevents uncontrolled proton leakage, and thus reduces ROS generation [25,26]. Another source of ROS in yeast is the activity of NADPH oxidases, which produce O_2_^•−^, hydrogen peroxide (H_2_O_2_), and hydroxyl radical (^•^OH) during fermentative metabolism [27]. To determine the effect of FC on the total ROS content, we treated yeast cells overnight with 0.25, 0.5, or 1.0 mM of the compound and performed flow cytometry analysis after staining with H_2_DCFDA, which reacts with ROS and emits fluorescence. The results showed that FC treatment dose-dependently reduced the cellular ROS level in yeast, which was indicated by a decrease in fluorescence intensity by up to 20% in the case of cells treated with 1.0 mM FC as compared with untreated cells (Figure 5). 

Taken together, these findings suggest that FC enhanced oxidative metabolism and mitochondrial efficiency, while reducing the level of ROS in yeast cells. 

### 3.3. FC Exerts Both Protective and Regenerative Effects on Yeast Treated with H_2_O_2_

Glucose is preferentially used by budding yeast as a carbon source, and in its presence, energy is mainly produced through alcoholic fermentation. The metabolic switch to oxidative phosphorylation occurs when glucose is depleted, and nonfermentable compounds, such as ethanol, lactate, or glycerol, begin to act as carbon sources [28,29]. Taking this into account, we analyzed the influence of FC on yeast mitochondria in conditions favorable to oxidative metabolism. We used a medium containing glycerol (YPG) instead of glucose for our experiments.

MMP (ΔΨm) is a crucial parameter of mitochondrial function and quality. It influences various processes, including energy metabolism, the uptake and storage of ions and proteins, or redox status. Changes in ΔΨm are an indicator of pathologies that can lead to cell death. A sharp decline in MMP could trigger apoptosis, while a rise in MMP could enhance ROS generation. Thus, it is important for a cell to maintain appropriate ΔΨm levels [25].

**Figure 6:**
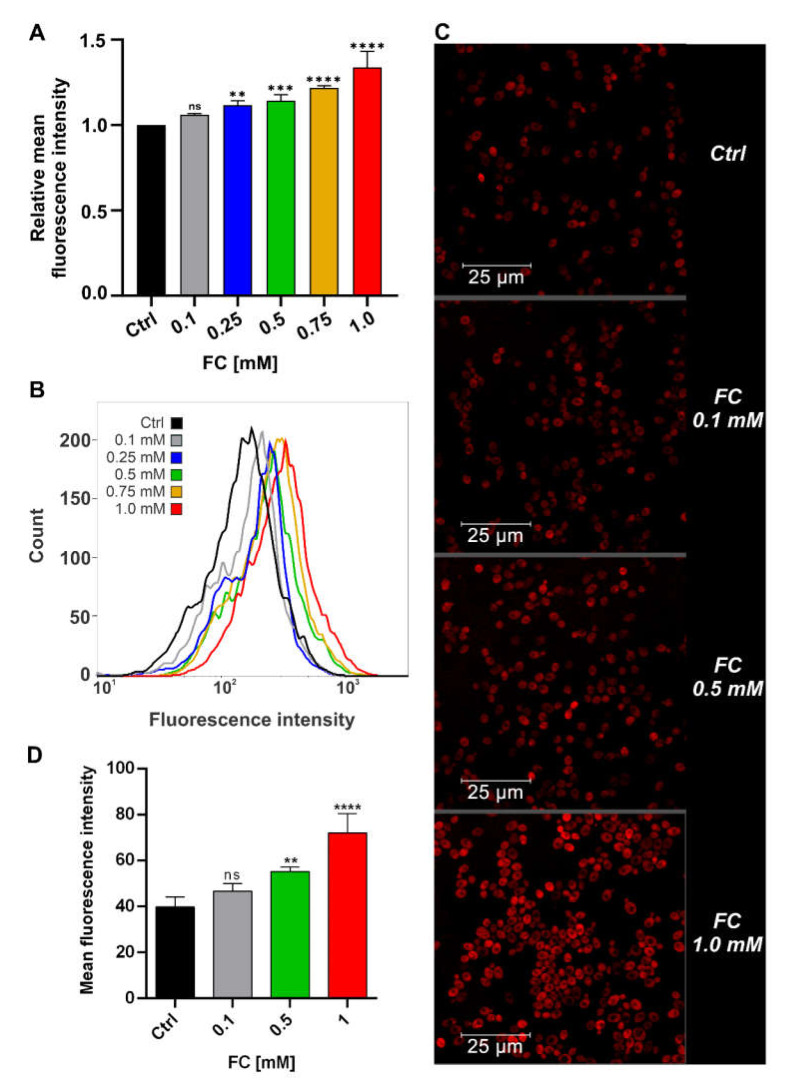
MMP (ΔΨm) changes in yeast cells growing in YPG medium supplemented with 0.1, 0.25, 0.5, 0.75, or 1.0 mM FC. (**A**,**B**) Flow cytometry analysis using TMRE staining. Bar graph (**A**) presents relative mean fluorescence intensity ± SD, whereas histogram (**B**) presents the data from at least three independent experiments. (**C**,**D**) Confocal microscopy analysis of ΔΨm using TMRE staining (ex/em: 543/570–610 nm). Representative images (**C**) and fluorescence intensity changes (**D**) in yeast cells treated with 0.1, 0.5, or 1 mM FC. Data are presented as mean fluorescence intensity ± SD of three independent experiments. Statistical significance versus control (ANOVA): (ns) non−significant, (**) *p* < 0.01, (***) *p* < 0.001, (****) *p* < 0.0001.

We evaluated the MMP of yeast cells grown overnight in YPG medium supplemented with FC via flow cytometry and confocal microscopy analysis using TMRE staining (Figure 6). TMRE, a cationic dye, targets active mitochondria and accumulates in proportion to ΔΨm. Flow cytometry analysis (Figure 6A,B) indicated a dose-dependent increase in MMP, ranging from approximately 5% for 0.1 mM (insignificant) to approximately 35% for 1.0 mM FC, and high mitochondrial energy status. The consistent results obtained from confocal microscopy imaging (Figure 6C,D) confirmed increased fluorescence intensity under the influence of FC.

**Figure 7:**
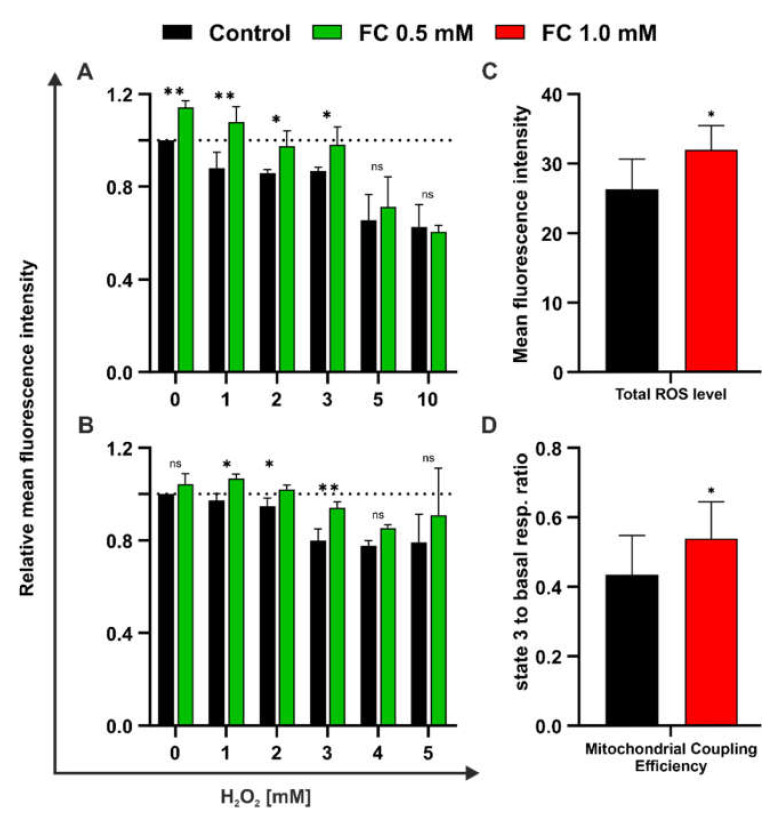
Effect of FC supplementation on yeast cells growing in medium containing glycerol. (**A**,**B**) MMP after H_2_O_2_ treatment measured with flow cytometry using TMRE staining. Data are presented as mean relative fluorescence intensity ± SD of at least three independent experiments. (**A**) Protective effect evaluated with H_2_O_2_ (1, 2, 3, 5, or 10 mM) treatment after overnight incubation of cells with 0.5 mM FC. (**B**) Regenerative effect evaluated with H_2_O_2_ (1–5 mM) treatment of cells followed by replacement of the medium with fresh YPG supplemented with 0.5 mM FC. (**C**) Flow cytometry analysis of ROS content in cells grown overnight in the presence of 1.0 mM FC using H_2_DCFDA staining. Data are presented as mean fluorescence intensity ± SD of three independent experiments. (**D**) Mitochondrial coupling efficiency evaluated using the Oxygraph+ system. Data are presented as the mean ratio of state 3 and basal respiration ± SD of three independent experiments. Statistical significance versus control (ANOVA: **A**,**B**; *t*-test: **C**,**D**): (ns) non−significant, (*) *p* < 0.05, (**) *p* < 0.01.

As mentioned above, elevated ΔΨm could cause an increase in the level of ROS. Therefore, we compared the cytoplasmic level of ROS in control cells and cells treated with the highest FC concentration with flow cytometry using H_2_DCFDA staining. We found that the ROS level was higher in cells treated with 1.0 mM FC (Figure 7C), which was reflected by approximately 15% more intensive fluorescence signal. However, no significant differences were noted in respiration parameters in YPG medium (Figure S1), except for mitochondrial coupling efficiency, which was about 10 percentage points higher in the case of yeast cells in the presence of FC (Figure 7D) and can indicate the enhanced load of the respiratory chain.

Finally, we investigated oxidative-stress-induced sensitivity in yeast cells by evaluating MMP loss after H_2_O_2_ treatment. We found that H_2_O_2_ exhibited a protective effect at a concentration of 1–3 mM (Figure 7A). In such a concentration range, cells cultured overnight in the presence of 0.5 mM FC showed not only significantly higher MMP, but their values were also equal to that of the untreated control. However, at higher H_2_O_2_ concentrations (5 and 10 mM), the effect of FC was not observed. A similar result was noted in the regenerative assay, in which yeast cells were treated for 1 h with H_2_O_2_ (1–5 mM) and then the medium was replaced with a fresh one supplemented with 0.5 mM FC (Figure 7B). Again, the positive effect of FC was evident with up to a 3 mM concentration of H_2_O_2_. In the case of 4 mM H_2_O_2_, the difference between FC-treated cells and control cells was not statistically significant, while for 5 mM H_2_O_2_, the results began to vary between the replicates and were difficult to interpret. 

Taken together, the findings suggest the positive effect of FC on yeast mitochondria, which was manifested by more effective oxidative phosphorylation and enhanced resilience to oxidative stress conditions. 

### 3.4. Effect of FC on the Expression Level of Genes Involved in Metabolism and Stress Resistance in Yeast Cells

**Figure 8:**
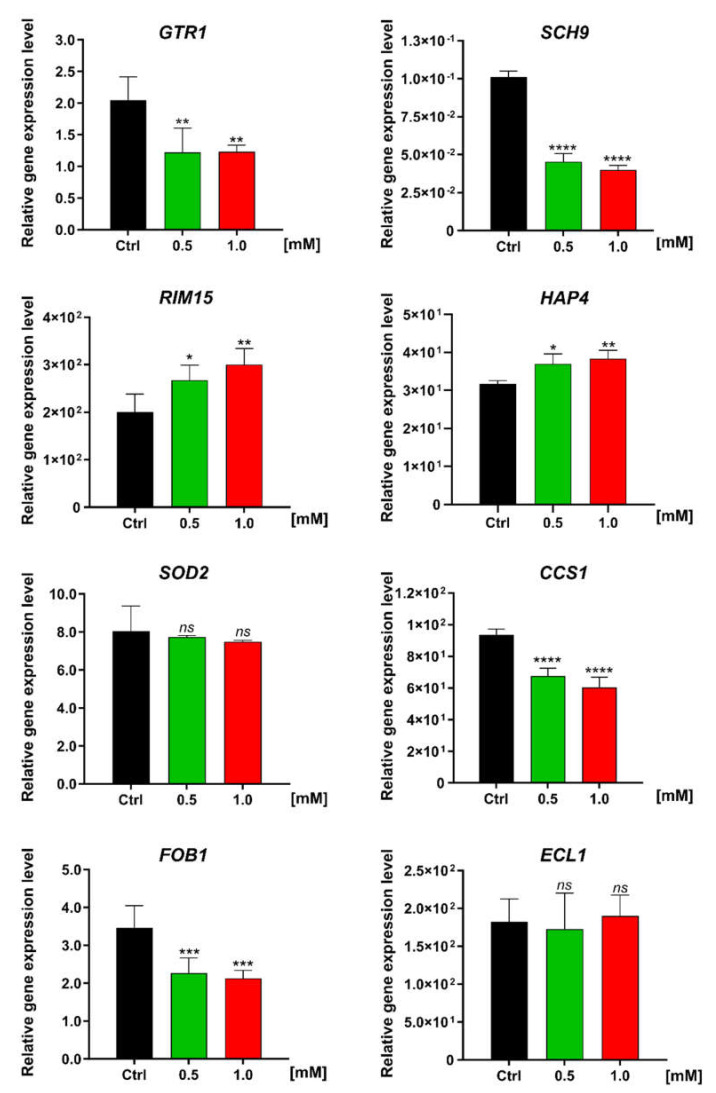
Changes in the expression of selected genes analyzed after treatment with 0.5 or 1.0 mM FC. Relative real−time PCR analysis was performed for the genes GTR1, SCH9, RIM15, HAP4, SOD2, CCS1, FOB1, and ECL1. Data are presented as mean ± SD of three biological replicates and three independent experimental repeats for each one. Statistical significance versus control (ANOVA): (ns) non−significant, (*) *p* < 0.05, (**) *p* < 0.01, (***) *p* < 0.001, (****) *p* < 0.0001.

As a final step, we investigated the effect of FC on the expression of several genes using real-time PCR (Table 3, Figure 8). The genes selected for the analysis included those involved in nutrient sensing and the TORC1 pathway (GTR1, SCH9, and RIM15), defense responses against oxidative stress (SOD2 and CCS1), the activation of mitochondrial respiration (HAP4) and the replication process (FOB1), and the extension of chronological lifespan (ECL1). 

We observed that FC had a diverse impact on the expression level of the genes associated with antioxidant defense. The expression of mitochondrial Mn superoxide dismutase 2 (SOD2) remained unchanged, despite the enhanced respiratory activity. However, the copper chaperone for Cu-Zn superoxide dismutase SOD1 (CCS1) was downregulated by over 25%. In yeast, CCS1 mediates cell maturation and is required for the activity of SOD1, a cytoplasmic dismutase which, apart from oxygen scavenging, is involved in the glucose repression of respiration [30,31]. In fact, we observed changes in the expression of genes involved in the TORC1 pathway, which promotes fermentative metabolism and rapid cell growth in response to nutrients. Under the influence of FC, the expression of the GTR1 gene, which is engaged in TORC1 activation, and the SCH9 gene, which codes its direct downstream kinase, was found to be markedly decreased. The change in GTR1 expression was almost twofold, while the expression of the SCH9 gene was more than twofold higher and the greatest compared to the expression of other analyzed genes. These findings suggest that FC strongly inhibited the TORC1 pathway. In the presence of FC, we observed the increased expression of RIM15, a gene that is suppressed by TORC1 and plays a crucial role in the nutrient deprivation response. We also observed a 10% increase in the expression level of the HAP4 gene, which is involved in the activation of mitochondrial metabolism and acts as a global regulator of respiratory gene expression. We also observed a considerable decrease (~40%) in the expression of FOB1, a gene involved in the generation of extrachromosomal rDNA circles (ERCs). The accumulation of ERCs in yeast cells is recognized as a hallmark of aging. However, we did not observe any change in the expression of the ECL1 gene.

## 4. Discussion

According to the World Health Organization (WHO), the number of people aged over 60 years will double by 2050, accounting for over 20% of the world’s population. In recent decades, the average lifespan has gradually increased, leading to an urgent need to improve healthspan [41]. The United Nations declared 2021–2030 as a “decade of healthy aging” in recognition of growing concern over aging society. The two main common age-related problems are sarcopenia and neurodegeneration, both of which are linked with mitochondrial dysfunction and the impairment of the redox state [42]. Indeed, in 1956, Harman proposed the free radical theory of aging, which states that aging is caused by cellular damage induced by free radicals produced over one’s lifetime [9]. The main source of free radicals in cells are mitochondria, which generate O_2_^•−^ during respiration and are therefore particularly prone to ROS damage. The phenomenon of age-associated mitochondrial dysfunction led to the proposal of the mitochondrial theory of aging (MFRTA) in the late 1970s [43]. Although it is now known that aging is a much more complex process that necessitates a more comprehensive explanation than free radicals alone [44], both aging theories are still regarded as important [45]. Despite the controversies surrounding the free radical theory of aging, ROS have been associated with age-associated frailty [46,47], as well as mitochondrial dysfunction [48,49].

Given the above, a number of compounds have been proposed as antiaging agents due to their antioxidant properties. However, antioxidant activity alone cannot extend lifespan and does not always adequately mitigate age-related dysfunctions [47]. The compound may show increased effectiveness when its antioxidant activity is extended with interactions with metabolic pathways, such as those associated with energy metabolism [46]. Since aging is a conserved process, many related pathways are common across species [50]. One of the most well-known is the target of rapamycin (TOR) signaling pathway, which promotes anabolic processes and cell growth in response to nutrients. A study on nematode *Caenorhabditis elegans* first linked aging with the TOR pathway [51], and to date, it is considered that the TOR pathway is the only target, the inhibition of which can prolong lifespan in all studied model organisms [52]. These organisms include yeast *S. cerevisiae*, in which the TORC1-Sch9 pathway plays a central role in regulating CLS, and its inhibition by rapamycin causes a significant increase in viability [34]. Another well-described antiaging intervention, calorie restriction (CR), also improves viability in a variety of species, and its action involves the partial inhibition of the TOR pathway [35,53]. Despite certain similarities, the exact mechanism of action of CR and rapamycin differs [54,55], but it is known that metabolism changes are a key factor in the aging process. 

It should be pointed out that budding yeast serves as a successful model organism in aging research. It possesses a short lifespan and is characterized by conserved aging-associated pathways and a similar stress response to higher eukaryotes. Furthermore, yeast represents a whole, unicellular organism in in vivo conditions. However, it has some limitations, such as a lack of a tissue-specific response and the absence of a cell wall. So far, multiple antiaging interventions have been examined in yeast. Apart from rapamycin and CR, resveratrol, metformin, or spermidine, which display promising activity in humans, have been tested in yeast [17]. It should be mentioned that, due to their antioxidant properties, flavonoids, a profound group of compounds, have been proven to have a positive effect on the aging of yeast [55].

Due to the advantages of the yeast model, we employed *S. cerevisiae* strain BY4741 and performed several experiments to evaluate the antiaging properties of FC. As the first step, we assessed the CLS of yeast in YPD medium. Synthetic defined media are mostly used in studies for this purpose; however, we used complete media, such as YPD, which are rich in all nutrients, including amino acids or nucleotides, and can mimic the conditions observed in higher eukaryotes [56,57,58,59]. The obtained results clearly indicated that FC has the potential to extend the viability of yeast cells (Figure 2A). We investigated the effect of FC in all applied concentrations and found that none of them are toxic or inhibit cell growth in the stationary phase (Figure 2B). A similar effect has been observed for supplementation with *Rhodiola rosea* extract, which resulted in prolonged CLS and no adverse effect on growth rate [60]. Interestingly, while CR-mediated lifespan extension also does not affect growth during the exponential phase, treatment with rapamycin slightly inhibits growth [61]. 

Since the aging process is highly related to mitochondria and changes in metabolism, we investigated the effect of FC on the activity of mitochondria. Previous reports on aging clearly show that decreased respiration leads to an increase in the level of ROS and reduces lifespan [62,63], and most antiaging compounds act by preventing the loss of mitochondrial function and accumulation of ROS [55]. Additionally, both rapamycin and CR increase the rate of oxygen consumption by yeast cells [61]. In line with those reports, we observed that FC prevented MMP loss in yeast cells during aging (Figure 3A) and increased the rates of respiration (Figure 4), accompanied by a reduction in ROS levels (Figure 5). These results are consistent with that of Barros, who showed that higher respiratory activity decreases the release of mitochondrial ROS and increases lifespan in *S. cerevisiae* [64]. Studies on human cells have shown that enhanced SRC is associated with a readiness to meet an extra energy demand and reduced ROS generation [19,65]. Recently, flavonoids from sacred lotus stamen extracts were shown to extend CLS by maintaining MMP and reducing the level of ROS [66]. In another study, the authors observed that the deletion of NAD+ carriers prolonged CLS and improved respiration efficiency, which was accompanied by a reduction in ROS level [67], and a similar effect was caused by reduced TOR signaling [68]. Interventions that mitigate the loss of mitochondrial activity can effectively counteract the aging process [69,70]. The inhibition of proaging TOR signaling by CR or other approaches enhanced the respiration rate and expression of genes associated with mitochondrial metabolism [70,71]. A recent study revealed that upon CR conditions, MMP was increased, resulting in either ATP production and ROS reduction in respiratory mutants, or increased ROS generation in nonrespiratory mutants [72]. In our study, we observed that yeast grown in glucose medium supplemented with FC exhibited increased MMP and respiration rates, which led to a reduction in ROS level and CLS extension, similar to that observed with CR. On the contrary, with a nonfermentable carbon source, which in our case was glycerol, FC failed to further elevate mitochondrial activity, and increased MMP resulted in an increased ROS level (Figure 6 and Figure 7). These findings led us to assume that FC may act by mimicking CR. 

As mentioned before, CR is the only intervention that prolongs lifespan as well as healthspan in a wide range of organisms, from yeast to humans. However, because maintaining a CR lifestyle throughout the lifespan is nearly impossible, compounds that mimic CR seem to be promising for further analysis [73,74]. In yeast, CR inhibits TORC1 and RAS2 and also induces a metabolic switch toward respiration [75]. The proaging TORC1-Sch9 pathway is a key regulator of yeast CLS and inhibits the antiaging protein kinase Rim15, which is involved in the response to environmental stress such as heat shock, oxidative stress, or nutrient starvation [76]. Therefore, we examined the effect of FC on the expression level of selected genes associated with the TORC1 signaling pathway and ROS scavenging using real-time PCR (Table 3, Figure 8). We observed that the expression of the SCH9 gene was downregulated, which confirmed our assumption of reduced TORC1/Sch9 signaling. A previous study demonstrated that the deletion of the SCH9 gene enhances respiration, lowers the ROS level, and extends CLS [77]. Furthermore, yeast mutants SCH9Δ and TORC1Δ are smaller than wild-type cells [78,79], which was also confirmed by the measurements of yeast cell area obtained in the present study (Figure 3B). The same phenomenon has been observed in CR conditions [80,81]. In addition, we also observed a reduction in the expression of GTR1, which is an activator TORC1. GTR1 was shown to be negatively regulated upon nutrient starvation [82], and its deletion led to reduced activity of TORC1 [83]. In line with the established relationship between the TORC1-Sch9 pathway and Rim15 protein kinase, we observed the upregulated expression of the RIM15 gene. Rim15 plays a crucial role in extending lifespan through the deletion of TORC1 and Sch9 or under CR conditions [78,84]. Since Rim15 is involved, among others, in oxidative stress response, it could be surprising that we did not observe the elevated expression of antioxidant defense genes such as SOD2 and CCS1. However, this finding is consistent with previous reports indicating the unchanged [85] or lowered [86,87] expression of genes associated with ROS scavenging upon CR conditions. A reduction in antioxidant defense and cytosolic ROS level (Figure 5) may be explained by the fact that the TORC pathway suppresses mitochondria activity by favoring fermentation, causing the depletion of SRC. Reduced oxygen consumption by the respiratory chain increases the intracellular availability of oxygen, thereby leading to elevated ROS generation [88]. In fact, of all the analyzed respiratory parameters, we observed the most profound increase in state U and thus in SRC (Figure 4B,E). Intriguingly, SRC deficiency has been shown to contribute to neurodegeneration in Alzheimer’s disease [89]. As expected from respiration analysis, FC supplementation elevated the expression of the HAP4 gene, which is involved in the activation of respiration and is upregulated under CR conditions [85]. Interestingly, a recent study showed that HAP4 overexpression was accompanied by a reduction in extrachromosomal rDNA circle (ERC) generation, and the effect was enhanced by the deletion of the FOB1 gene [90], the downregulation of which was also noted in our study. It must be noted that the accumulation of ERCs in yeast mother cells is considered a hallmark of aging [37]. 

As mentioned before, with a nonfermentable carbon source (glycerol), FC increases MMP (Figure 6), but not respiration, resulting in elevated intracellular ROS levels (Figure 7C). This result corresponds to those obtained for nonrespiratory mutants cultivated under CR conditions [72]. The suppression of respiration enhancement via TOR inhibition on a medium containing glycerol as a carbon source has also been previously documented [68]. We believe that FC forces mitochondrial metabolism on both nonfermentable and fermentable carbon sources. However, since respiration is the main mechanism of energy metabolism in the case of nonfermentable carbon sources [91], it is maintained at a maximal level and FC fails to extend it further. This results in an overload of the respiratory chain, which was observed by increased MMP and the contribution of the phosphorylating state in basal respiration (Figure 7D), and further by elevated ROS level, in our study. Nevertheless, upon H_2_O_2_ treatment, FC-supplemented yeast cells do not suffer a sudden loss of MMP, and FC helps restore MMP after treatment with H_2_O_2_, which indicates its antioxidant properties. 

## 5. Conclusions

The present study investigated the antiaging properties of FC in yeast cells. The results showed that prolonged lifespan was associated with enhanced respiration level, an increase in MMP and its maintenance during lifespan, and reduced ROS level and activity of the TORC1-Sch9 pathway. Taking all of these results into consideration, including changes in the expression levels of selected genes, it can be concluded that the action of FC mimics CR condition. Yeast is a simple, single-cell organism, the metabolism of which differs from mammals. However, since CR is an antiaging intervention that is effective in various species, we consider FC as a promising compound with the potential to improve healthspan in other organisms as well. We will continue our research on FC to understand its activity in other aging models. 

## Figures and Tables

**Table 1:** Primer sequences used in the qPCR analysis.

Gene	Forward Primer (5′–3′)	Reverse Primer (5′–3′)	UPL
CCS1	TGAACCACCCAGAAAACGA	GCAATGACTCCCAGAAATGAG	69
ECL1	AATAAGTTATATTGCTCCGAAGATTGT	TTTTATGCAAGTGGGATAATAATTTCT	161
FOB1	CGAAACGTAAACCTGTGCAA	CTCTCTTAGTCTCAAACTTGGCATT	85
GTR1	AAGATGGATCTTGTTCAGTTGGA	CAGGTTTTTCATCATGATTTGG	17
HAP4	TCAGGATGAAAGCGCTGATT	AGCCAAAGTGATTTCTGAACCT	9
RIM15	TTTCCCTCCTTGATATTTCTCG	TCGAATTTGTGGGATTTGCT	63
SCH9	TTGGGATCTCACTCGGAATTA	GGATACAGTCTAACTTGGCCTAAGAA	44
SOD2	AAACCACTGTCTATTCTGGGAAA	CTCGTCGATTGCCTTTGC	36
ACT1 *	TTCCAGATGGTCAAGTCATCA	AAACAGAAGGATGGAACAAAGC	45
ALG9 *	CAAGAGCATGCTTAGGCTTTTT	CCCGGATTAAACAATTGGAA	27
TAF10 *	GAGGAGATTCTAGAGATGATGGACA	AGTCTATTACTGCATCGGGAATG	63
TFC1 *	CTCAAATGCCATAGAGGAGGA	TGGCGCCATTATCATCAAA	9
UBC6 *	AGGCTCACAAGAGATTGACGA	GGGCGAGCAAGAATATATGG	31

* Reference genes.

**Table 2:** Effect of FC supplementation on yeast CLS. Mean percentage of live cells on the following days of CLS assay. Data are presented as mean ± SD from three independent experiments. Statistical significance versus control (ANOVA): (*) *p* < 0.05, (**) *p* < 0.01, (****) *p* < 0.0001.

Day of CLS		FC (mM)		
	Ctrl	0.25	0.5	1.0
3	99.4 ± 0.001	99.6 ± 0.007	99.7 ± 0.002	100.0 ± 0.000
5	98.1 ± 1.285	98.8 ± 0.957	98.4 ± 1.603	98.8 ± 1.341
7	93.9 ± 3.893	96.3 ± 2.813	95.9 ± 3.553	98.3 ± 1.192
10	92.1 ± 2.616	92.1 ± 2.616	93.3 ± 2.951	96.2 ± 1.259
12	81.9 ± 6.879	84.6 ± 9.620	88.0 ± 8.443	91.5 ± 3.888 *
14	74.1 ± 1.401	81.7 ± 6.798	84.5 ± 7.386 *	90.5 ± 1.050 ****
17	62.6 ± 6.130	73.5 ± 5.770 **	80.8 ± 6.503 ****	88.8 ± 0.656 ****
19	53.1 ± 14.623	70.6 ± 2.512 ****	73.2 ± 4.595 ****	81.1 ± 0.403 ****

**Table 3:** List of genes analyzed using real-time PCR with the description of the function of their products. Upward arrow (upregulated expression); downward arrow (downregulated expression); the number of arrows correlates with the strength of expression change.

Cellular Significance	Gene		Changes in Expression Level	Function
Antioxidant defense	CCS1	Copper chaperone for SOD1	↓↓	Involved in oxidative stress protection; increase in protein abundance in response to DNA replication stress [32]
	SOD2	Superoxide dismutase	No change	Mitochondrial manganese superoxide dismutase; protects cells against oxygen toxicity and oxidative stress [32]
Nutrient sensing and TORC1 signaling pathway	GTR1	GTP-binding protein resemblance	↓↓↓	GTPase that activates TORC1 in response to amino acid stimulation [33]
	RIM15	Regulator of IME2	↑↑	Protein kinase involved in cell proliferation in response to nutrients; plays a crucial role in the entry of cells into the stationary phase upon nutrient starvation conditions [34,35,36]
	SCH9	Serine/threonine protein kinase	↓↓↓	Direct downstream protein kinase of TORC1 [34,35,36]
Replication process	FOB1	Fork blocking less	↓↓	Required for replication fork blocking; involved in ERCs generation [37]
Respiratory activity	HAP4	Heme activator protein	↑	Transcriptional activator and global regulator of respiratory gene expression; plays a central role in shifting cells from fermentative to respiratory growth [38,39]
Unknown function	ECL1	Extender of the chronological lifespan	No change	Mitochondrial-dependent role in the extension of CLS; unknown exact molecular function [40]

## Data Availability

All of the data is contained within the article and the supplementary materials.

## Supplementary Materials

The following supporting information can be downloaded at: https://www.mdpi.com/article/10.3390/antiox11050850/s1. Figure S1: Effect of FC supplementation on the energy status of yeast cells growing in YPG medium.
