# Supplementary material for: Antiaging Effect of 4-N-Furfurylcytosine in Yeast Model Manifests through Enhancement of Mitochondrial Activity and ROS Reduction
Source: Antioxidants (Basel). 2022 Apr 26;11(5):850. doi: 10.3390/antiox11050850 (PMC9137487; doi:10.3390/antiox11050850)
Supplement: Supplementary file 1 [file antioxidants-11-00850-s001.zip › Supplementary data.pdf]

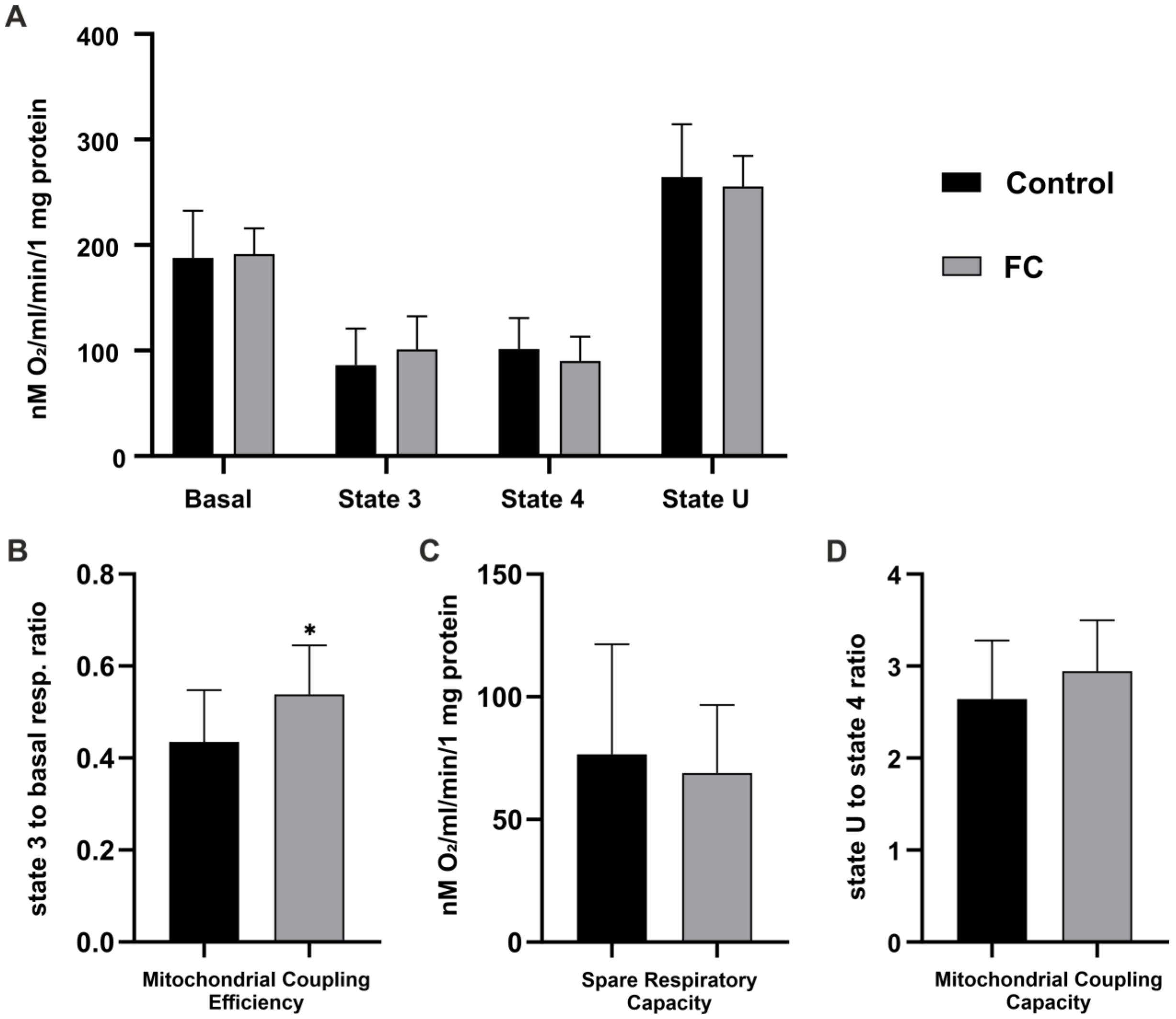

Figure S1. Effect of FC supplementation on the energy status of yeast cells growing in YPG medium. The recording was performed in YPG medium by using the Oxygraph+ system. (**A**) Respiratory states calculated for control (black bars) and treated (gray bars) yeast cells. (**B**) Contribution of state 3 to basal respiration, which corresponds to mitochondrial coupling efficiency. (**C**) Spare respiratory capacity (SRC) is the reserve between basal and maximal respiration. (**D**) Mitochondrial coupling capacity was calculated as state U and state 4 ratio. The data are presented as mean ± SD of three independent experiments. Statistical significance versus control (t-test): (*) $p < 0.05$
